# Epidemiological characteristics of acute disseminated encephalomyelitis in Nanchang, China: a retrospective study

**DOI:** 10.1186/1471-2458-14-111

**Published:** 2014-02-04

**Authors:** Chang-hui Xiong, Yan Yan, Zhen Liao, Shi-hui Peng, Hai-rong Wen, Yan-xia Zhang, Shu-hua Chen, Jian Li, Hai-ying Chen, Xiao-wu Feng, Hui-qing Yao, Li Huang, Li Zhang

**Affiliations:** 1 School of Public Health, Central South University, Changsha 410078, China; 2 Nanchang City Center for Disease Control and Prevention, Nanchang, China; 3 Donghu District Center for Disease Control and Prevention, Nanchang, China

**Keywords:** Acute disseminated encephalomyelitis, Incidence, Epidemiology

## Abstract

**Background:**

Acute disseminated encephalomyelitis (ADEM) is an autoimmune disease that typically follows a monophasic course and may affect any age group. The precise population-based incidence of ADEM is still unknown in most countries. In China, there is no ADEM surveillance system. The exact incidence of ADEM is difficult to estimate, and other epidemiological characteristics of ADEM are unknown. The purpose of this study is to investigate the epidemiological characteristics of ADEM in Nanchang, China.

**Methods:**

A retrospective investigation was conducted with ADEM patients admitted to second-level and third-level hospitals in Nanchang from 2008 to 2010, aiming to analyse the epidemiologic characteristics of ADEM in the population in Nanchang. ADEM patients, defined as patients who were diagnosed according to the consensus definition of ADEM provided by the International Pediatric MS Study Group, were enrolled in the study. The data were extracted from the ADEM patients’ medical records.

**Results:**

Forty-seven ADEM patients were investigated. The average annual incidence was 0.31/100,000; the incidence among males (0.31/100,000) was nearly equal to that among females (0.31/100,000). The median age of onset was 25.97 years old, and the peak incidence was observed in the 5- to 9-year-old age group (0.75/100,000), followed by the over-60 age group (0.55/100,000). ADEM occurs throughout the year, but it occurs most frequently in March (n = 7) and least frequently in April and July (both n = 2). The patient numbers are roughly even in the other months. In the 2 months before the onset of ADEM, 15 patients presented with a preceding infection, but none of the patients received a vaccination. An increased number of vaccination was not accompanied by a corresponding increased number of cases of ADEM.

**Conclusions:**

The average annual incidence of ADEM was 0.31/100,000 in Nanchang. The incidence among males was nearly equal to that among females. The peak age of onset was 5–9 years old. The peak season of onset was not apparent. There was no evidence of an association between increased number of vaccines administered and number of cases of ADEM in Nanchang, China.

## Background

Acute disseminated encephalomyelitis (ADEM) is a disease that is mainly characterised by an inflammatory reaction and demyelination of the central nervous system (CNS) [1]. ADEM, which typically follows a monophasic course, may occur in any age group, especially in children, but cases in children under 3 years old are rarely reported. It usually occurs in winter and has no unified diagnostic criteria [2-5]. Some researchers have noted that the incidence rates of ADEM are not different between genders, races, and regions [6,7]. The pathogenesis of ADEM is not fully known, but adhesion molecules, chemokines, matrix metalloproteinase, and other cell factors can play important roles in its occurrence and development [8].

ADEM usually occurs following an infection (mainly exanthematous disease) or vaccination (e.g., rabies, small pox or measles vaccines) [4,9] and primarily affects the white matter of the brain and the spinal cord [10,11]. The precise annual population-based incidence is unknown, but it is estimated to be 0.8/100,000 [12]. The mean age of onset ranges from 5 to 8 years old [13,14]; the incidence of ADEM in children is higher than in adults. Older patients are rarely reported [15]. There is no clear research on the global distribution of ADEM. In Fukuoka Prefecture, Japan, the annual incidence of ADEM in children under 15 years old was 0.64/100,000, the mean age of onset was 5.7 years old, and the incidence in males was 2.3 times higher than in females [16]. ADEM occurs more frequently in the winter and in the spring.

In China, there has been little research about the incidence of ADEM, and the exact incidence of ADEM is still difficult to estimate. To understand the epidemiologic characteristics of ADEM in Nanchang in Jiangxi Province, China, a retrospective investigation of the ADEM patients admitted to all hospitals in 2008–2010 was performed at the end of 2011.

## Methods

Jiangxi is situated in central China, and Nanchang, the capital city, is the centre of Jiangxi politics, economy, cultures and health services. There is a population of 4,981,054 in Nanchang (average from 2008 to 2010). In the Chinese health service system, hospitals are divided into three levels. The higher-level hospitals have greater medical service capabilities. First-level hospitals are basic hospitals and primary health care institutions that mainly provide primary prevention services to community residents or referrals for serious or rare diseases. The second-level hospitals are regional hospitals and regional medical prevention technology centres. Their main functions include disease treatment, acceptance of referrals and technical guidance for medical services provided by primary hospitals. Third-level hospitals are general hospitals with the greatest ability to provide comprehensive medical treatment and to engage in teaching and scientific research, especially treatment of serious and rare diseases.

There are 35 second-level and third-level hospitals in Nanchang. Of these, 16 hospitals are third-level hospitals with the highest level of medical services in Jiangxi; they possess over 15,000 beds and provide advanced medical services. The 19 remaining hospitals are second-level hospitals. There are 263 first-level hospitals, which provide lower-level medical services. Most first-level hospitals do not even have medical beds, so they are incapable of diagnosing and treating severe illnesses (e.g., ADEM). Severely ill patients who are admitted there will be transferred to the second-level or top-level hospitals. Therefore, the patients admitted by second–level and third-level hospitals comprise all the cases of ADEM in Nanchang and reflect the actual incidence of ADEM in Nanchang.

We approached and sought help from local health administration authorities (i.e., Jiangxi Provincial Health Department and Nanchang City Health Bureau) in advance, so the study was conducted successfully. We investigated all ADEM cases admitted by the 35 second-level and third-level hospitals in Nanchang from 2008 to 2010. All the cases were identified from the electronic medical records information management system (EMRIMS) in each hospital using the consensus definitions of ADEM that were proposed by the International Pediatric MS Study Group. The definition of ADEM was based on the criteria described below [17].

The infection symptoms appear first. It occurs acutely or subacutely and indicates that an illness is severe or dangerous.

With regard to the clinical presentation, the brain or spinal cord usually suffers multifocal damage. The cerebral symptoms are predominantly mental symptoms, disturbances of consciousness and meningeal irritation; pyramidal tract and cerebellar signs can appear. The myelopathic symptoms include paraplegia, rising palsy and urinary disorders, etc.

The cerebrospinal fluid (CSF) pressure is normal or higher, CSF-monocytes rise, immunoglobulin G rises, and the electroencephalo-graph is abnormal. Computed tomography (CT) and magnetic resonance imaging (MRI) show scattered lesions on the brain and spinal cord.

Professional and technical personnel manually searched the cases using the same criteria without EMRIMS, and then a senior neurologist checked the search results. The investigator collected relevant data from the medical records of the searched cases and supplemented some data at the grassroots level using vaccination records and information from the patients’ family members. During the investigation, if the same case was diagnosed and cured many times in the same hospital, the results depended on the hospitalisation records and the discharge diagnoses. If the same case was diagnosed and cured in different hospitals, the results depended on the medical records and discharge diagnosis at the upper-level hospital. The same patients who were seen repeatedly were considered new cases each time.

### Data collection

The following data were extracted from the patients’ medical records: gender, date of birth, residential area, rural or urban residence, occupation, marital status, date of onset and allergic history, family medical history, childbirth history, and disease history within 2 months before the onset of ADEM.

The data on the total population of Nanchang from 2008 to 2010 were acquired from the Nanchang City Statistic Bureau. The vaccination data for Nanchang from 2008 to 2010 were obtained from the Nanchang City Center for Disease Control and Prevention.

### Data analysis

The survey data were input into Chinese version EpiData 3.1 (270108) and Microsoft Office Excel 2003s. IBM SPSS 20 was used to check, sort and analyse the data and to calculate the age- and gender-adjusted incidence rates in the total population of Nanchang. The incidence rates were compared by year, gender and composition ratio using the chi-square test. The assumed distribution of a single sample was tested with the Kolmogorov-Smirnov test. All of the tests were two-sided, and 0.05 was the level of significance.

### Ethics statement

This investigation was ratified by the Ethics Committee of Nanchang City Center for Disease Control and Prevention (Reference number 2011002). The participants wrote Informed Consent. The relevant data were extracted from the medical records, and no case specimens were collected. All the data were analysed anonymously. The patients’ private information was safeguarded effectively.

## Results

Forty-seven cases of ADEM were identified and investigated: 44 from third-level hospitals and 3 from second-level hospitals. The average annual incidence was 0.31/100,000 in 2008–2010 (Table 1). The incidence rates of ADEM changed insignificantly from year to year. There were as many male cases as female cases, with no significant difference between the genders. The incidence among males (0.31/100,000) was almost equal to the incidence among females (0.32/100,000), with no significant difference.

**Table 1:** Overall incidence and gender-based incidence of ADEM in Nanchang

**Year**	**Male**	**Female**	**Total population**	**Male case**	**Female case**	**Total case**	**Incidence**	**(1/100,000)**	
	**(n)**	**(n)**		**(n)**	**(n)**	**(n)**	**In males**	**In females**	**Overall**
2008	2,599,218	2,348,048	4,947,266	11	5	16			
2009	2,607,689	2,365,636	4,973,325	7	7	14	0.27	0.30	0.28
2010	2,625,446	2,397,124	5,022,570	6	11	17	0.23	0.46	0.34
Sum	7,832,353	7,110,808	14,943,161	24	23	47	0.31	0.32	0.31
Chi-square				3.701			0.034		0.276
*P*				0.157			0.853		0.871

The mean age of onset was 33.40 years old (95% confidence interval (CI) 26.34 to 40.45), and the median age was 25.97 years old. Table 2 indicates the incidences in different age groups. The incidence was the highest in children 5 to 9 years old (0.75/100,000), followed by individuals over 60 years old (0.55/100,000), but there were no significant differences between the age groups (χ^2^ = 15.392, *P =* 0.052). The average annual incidence was 0.47/100,000 in children under 15 years old (n = 16) and 0.27/100,000 in those over 15 years old; the difference between children and adults was not significant (χ^2^ = 3.460, *P =* 0.063).

**Table 2:** Age-based incidence of ADEM in Nanchang

**Age group**	**Population**	**Case**	**Average annual incidence**
	**n**	**n**	**(1/100,000)**
0-	1,054,987	3	0.28
5-	1,069,930	8	0.75
10-	1,264,191	5	0.40
15-	1,129,703	3	0.27
20-	1,673,634	5	0.30
30-	2,665,860	2	0.08
40-	2,359,525	6	0.25
50-	1,921,691	5	0.26
60-	1,803,640	10	0.55
Total	14,943,161	47	0.31

Of the 47 patients, 31 patients were over 15 years old, and 3 patients were under 3 years old.

There are 56 nationalities in China. The Han nationality is the most common. All 47 of the patients were the Han nationality. With regard to occupation, students (from university and schools) (n = 13) and peasants (n = 13) were the most common roles. Of the 31 patients older than 15 years old, 26 (83.87%) were married.

**Figure 1:**
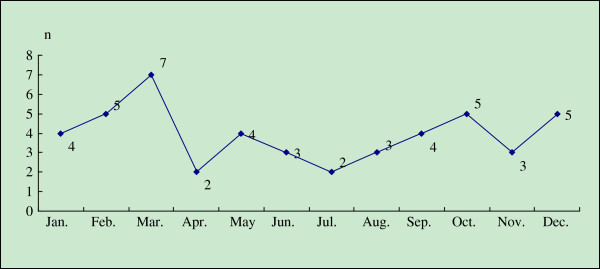
Monthly distribution of the onset of the 47 ADEM cases.

The 47 patients were distributed uniformly throughout the year in terms of the onset time (*P =* 0.188). Figure 1 indicates that the peak month of onset was March (n = 7, 14.89%), and the nadir month of onset was April (n = 2) or July (n = 2). In the comparison of the onset time distributions between the genders, the expected values for all the cells were less than 5, so the 12 months were divided into four quarters (January to March, April to June, July to September, and October to December). Then, 16, 9, 9 and 13 of the 47 cases were included in the first, second, third and fourth quarters, respectively. However, 50% of the expected cell values were less than 5 when a comparison was made between the genders, and 83.3% of the expected cell values were less than 5 when a comparison was made between years.

Table 3 shows whether the patients fell ill or not within two months before the onset of disease. Fifteen patients had a history of preceding illness. There was no significant difference between the genders (χ^2^ = 0.170, *P =* 0.680) or between the years (χ^2^ = 2.717, *P =* 0.257). In this study, all 15 patients who fell ill within 2 months before the onset (6 cases: < 14 years old; 5 cases: 15–59 years old; 4 cases: > 60 years old) had a history of preceding infection and had symptoms of a respiratory tract infection. Among the 15 patients, 7 patients were given an antigen antibody test. One patient had the Epstein-Barr virus and Coxsackie virus, 2 patients had cytomegalovirus, 1 patient had cytomegalovirus and herpes simplex virus, 1 patient had Gram-positive bacteria, and three patients received negative results. Among the 15 patients, 2 patients had co-morbid chronic bronchitis, 1 patient had severe pneumonia, and 1 patient had high blood pressure.

**Table 3:** Falling ill or not within 2 months before the onset of ADEM

	**Male**	**Female**	**2008**	**2009**	**2010**
being ill	7	8	7	5	3
Not being ill	17	15	9	9	14
Total	24	23	16	14	17

The study showed that no patients were vaccinated within two months before the onset of disease.

The results of ecological study on the relationship between vaccines and ADEM are shown below. The situation of immunisation vaccine campaigns in Nanchang during 2008–2010 were as follows: in 2008, there were 1,535,873 routine immunisation doses administered to the total population; in 2009, a supplementary immunisation campaign for the measles vaccine and a revaccination campaign for the hepatitis B vaccine for children under 15 years old were conducted, and there were 3,686,817 total vaccination doses; in 2010, a supplementary immunisation campaign for the measles vaccine for children under 4 years old, a revaccination campaign for the oral poliovirus vaccine, and a revaccination campaign for the hepatitis B vaccine for children under 15 years old were conducted for a total of 3,274,689 doses. The number of vaccination administered in 2009 was 2.40 times higher than the number administered in 2008, while the number of ADEM patients decreased by 2. The number of vaccination administered in 2010 was 2.13 times higher than the number administered in 2008, but the number of ADEM patients in 2010 was only one more than the number in 2008.

## Discussion

This study was the first large-scale investigation of ADEM incidence in Nanchang, Jiangxi in China, and it could help us to understand the baseline ADEM incidence in Nanchang. The investigation showed that the average annual incidence of ADEM in Nanchang was 0.31/100,000, which is slightly lower than the incidence in San Diego between 1991–2000 (0.4/100,000), than the incidence among children with demyelination disease in Thailand between 1997–2006 (4.1/100,000) [18], and then an estimation of 0.8/100,000 [12]. However, the incidence was higher than the incidence among Germans (0.07/100,000) [19]. These results indicate that the incidence rates of ADEM differ among countries, most likely due to the differences in race, environment or climate.

In the population in Nanchang, the average age of onset was 33.40, and the median age was 25.97. The incidence was 0.55/100,000 among those over 60 years old, 0.47/100,000 among those under 14 years old, and 0.27/100,000 among those 15–59 years old. The incidence rate was 0.31/100,000 among males and 0.32/100,000 among females. Reportedly, ADEM mainly occurs in children younger than 10 years old [3,4] or mainly in children and young people [20]. Previous studies on ADEM have focused on children but rarely on adults. Among the children with demyelination disease in Thailand between 1997 and 2006, the incidence rate was 4.1/100,000, the average age of onset was 6.9 years old, and most of the patients presented with a preceding infection [18]. In Germany, the incidence rate of ADEM was 0.09/100,000 among children under 10 years old and 0.03/100,000 among children 10–15 years old. Thus, children 3–8 years old were the peak group of onset; the incidence rate was insignificantly higher in males than in females [19]. Banwell reported that ADEM mostly attacked children under 10 years old, but the incidence rates did not differ between the sexes [21], and the median age of onset was 6.5 [1]. The results of our study are similar to some other studies in the literature: ADEM may occur in any age group, and the incidence rate is insignificantly higher among children under 14 years old than among people older than 15 years old. The incidence rate is the highest among children 5–9 years old (0.75/100,000), followed by individuals older than 60 years old (0.55/100,000). Senile ADEM has rarely been reported [15], which attracts our attention.

ADEM occurs most frequently in March (n = 7) and least frequently in April or July (both n = 2). It is easier for the residents to become infected with a precursor disease, such as a respiratory tract infection, because of the winter weather in Nanchang. In the northern hemisphere subtropical zone, it is coldest in January and February. In addition, the Chinese spring festival, the most important traditional festival in China, is often around February. People are often reluctant to go to hospital until spring festival is over. Thus, the number of patients may increase in March. When the 12 months were divided into 4 seasons, the incidence rates did not differ by season. This conclusion is slightly different from the conclusion that ADEM occurs more frequently in the spring and in the winter [6,22]. Preceding infections generally occur 2 to 60 days before the onset of ADEM [3,23]; two-thirds of the child patients had a preceding infection, one-sixth of the child patients had a history of inoculation [16], and approximately 50% of the adult patients had no preceding infection. The viruses that cause infection mainly include measles, parotitis, urticaria, varicella-zoster virus, cytomegalovirus (CMV), Epstein-Barr virus, herpes simplex virus, hepatitis A or B, Coxsackie virus, influenza A or B, HIV, human T-cell lymphotropicvirus-1 (HTLV-1), human herpes virus 6 [24], and the bacteria are mainly pneumonia mycoplasma [25]. In this study, 15 patients (31.92%) had other diseases before the onset of ADEM (all were preceding infections); 6 patients under 14 years old (37.5%) and 4 patients older than 60 years old (40%) had preceding infections. The detected rates of infection with pathogens were consistent with previous studies.

ADEM often occurs after vaccination, so it is believed that ADEM related to inoculation most likely occurs because the inoculation induces autoimmune disorders that cause brain and spinal cord immune injuries. Inoculation with vaccines against hydrophobia (in particular), smallpox or measles [26], poliomyelitis [27], type-B encephalitis, hepatitis B, pertussis, or influenza can cause ADEM, but cases caused by the influenza vaccine are rarely reported [24,28]. With vaccination, the total incidence rate of ADEM was 0.1-0.2/100,000 [12], lower than the incidence rate after infection with the measles virus (1:1,000) [29]. The incidence of ADEM was 1:1,000 - 1:20,000 after inoculation with the measles vaccine and 1:7,000-1:50,000 after inoculation with the rabies vaccine [30]. Only one of the 14.3 million inoculators in Sichuan, China in 2007–2008 had ADEM. The results of the study showed that no patients received a vaccination within 2 months before the onset of ADEM. In addition, the results of ecological study showed that an increased number of vaccination was not accompanied with a corresponding increased number of cases of ADEM. Because ecological study has some main limitations including ecological fallacy and difficult control of some confounding factors etc. The results of ecological study cannot provide strong evidence for an association between exposed factors and onset of the disease. Thus, the authors inferred discreetly the following conclusion with the existing research data: there was no evidence of an association between increased number of vaccines administered and number of cases of ADEM in Nanchang, China. It would be satisfactory if a cohort study on the association between vaccine and ADEM was performed.

In this study, the authors searched all the hospitals that could receive and cure ADEM patients, but some ADEM patients might not go to hospital. Some ADEM patients might have been missed, so the incidence of ADEM might be underestimated. The information in the medical records might be incomplete, and information bias might be present.

## Conclusions

The average annual incidence of ADEM was 0.31/100,000 in Nanchang. The incidence rates were nearly equal between genders. The high-risk age group is 5–9 years old. There was no seasonal variation in the incidence of ADEM. There was no evidence of an association between increased number of vaccines administered and number of cases of ADEM in Nanchang, China.

## Competing interests

The authors declare that they have no competing interests.

## Authors’ contributions

YY designed the study, and CX prepared the manuscript. SP performed the statistical analysis and drafted the article. ZL, HW, YZ, SC, JL, HY, LH and LZ participated in and conducted the fieldwork. HC and XF coordinated the study. All the authors read and approved the final manuscript.

## Pre-publication history

The pre-publication history for this paper can be accessed here:

http://www.biomedcentral.com/1471-2458/14/111/prepub
